# A cardiac mitochondrial cAMP signaling pathway regulates calcium accumulation, permeability transition and cell death

**DOI:** 10.1038/cddis.2016.106

**Published:** 2016-04-21

**Authors:** Z Wang, D Liu, A Varin, V Nicolas, D Courilleau, P Mateo, C Caubere, P Rouet, A-M Gomez, G Vandecasteele, R Fischmeister, C Brenner

**Affiliations:** 1 INSERM UMR-S 1180, Faculté de Pharmacie, Université Paris-Sud, Université Paris-Saclay, Châtenay-Malabry, France; 2 UMS–IPSIT, Université Paris-Sud, Université Paris-Saclay, Châtenay-Malabry, France; 3 INSERM I2MC, UMR 1048, Université Paul Sabatier, Toulouse, France

## Abstract

Although cardiac cytosolic cyclic 3′,5′-adenosine monophosphate (cAMP) regulates multiple processes, such as beating, contractility, metabolism and apoptosis, little is known yet on the role of this second messenger within cardiac mitochondria. Using cellular and subcellular approaches, we demonstrate here the local expression of several actors of cAMP signaling within cardiac mitochondria, namely a truncated form of soluble AC (sAC_t_) and the exchange protein directly activated by cAMP 1 (Epac1), and show a protective role for sAC_t_ against cell death, apoptosis as well as necrosis in primary cardiomyocytes. Upon stimulation with bicarbonate (HCO_3_^−^) and Ca^2+^, sAC_t_ produces cAMP, which in turn stimulates oxygen consumption, increases the mitochondrial membrane potential (ΔΨm) and ATP production. cAMP is rate limiting for matrix Ca^2+^ entry via Epac1 and the mitochondrial calcium uniporter and, as a consequence, prevents mitochondrial permeability transition (MPT). The mitochondrial cAMP effects involve neither protein kinase A, Epac2 nor the mitochondrial Na^+^/Ca^2+^ exchanger. In addition, in mitochondria isolated from failing rat hearts, stimulation of the mitochondrial cAMP pathway by HCO_3_^−^ rescued the sensitization of mitochondria to Ca^2+^-induced MPT. Thus, our study identifies a link between mitochondrial cAMP, mitochondrial metabolism and cell death in the heart, which is independent of cytosolic cAMP signaling. Our results might have implications for therapeutic prevention of cell death in cardiac pathologies.

Mitochondria are involved in cell life and fate decision through their multiple biological functions in energetic metabolism, reactive oxygen species (ROS) detoxification and cell death.^1, 2, 3^ These functions are crucially regulated to provide sufficient energy for cell functions, maintain mitochondrial membrane integrity and avoid excessive cell death.^4, 5^ Moreover, mitochondria may participate in Ca^2+^ homeostasis via matrix Ca^2+^ accumulation through the mitochondrial Ca^2+^ uniporter (MCU), Ca^2+^ release into the cytosol and propagation to other mitochondria, notably in excitable cells.^6, 7, 8^ In cardiomyocytes, intracellular Ca^2+^ movements are crucial for proper myofibril contraction and relaxation and energetic metabolism. Moreover, recent studies in cardiomyocyte-specific mutant mouse lacking the MCU showed a link between mitochondrial Ca^2+^ uptake and energetic supply in relation with cardiac workload during acute stress.^9, 10^ In contrast, excessive mitochondrial Ca^2+^ accumulation, ROS production and adenine nucleotide depletion result in the sudden opening of a megachannel, namely the permeability transition pore complex. The prolonged opening of this unspecific pore leads to the mitochondrial permeability transition (MPT), cell death, inflammation and irreversible tissue damage.^11, 12^ MPT can be a critical event in severe cardiac diseases such as ischemia–reperfusion injury and heart failure (HF) as well a radiation-induced cardiotoxicity.^11, 13, 14^ Hence, MPT inhibition by cyclosporin A (CsA) has been shown to limit cardiac damages and improve cell survival. Inhibition of MPT has thus become an attractive therapeutic strategy in cardioprotection.^15^

Cyclic 3′,5′-adenosine monophosphate (cAMP) is a major second messenger in many organs, particularly in the heart, where it regulates diverse physiological processes such as Ca^2+^ homeostasis, beating frequency and myocardial contractility as well as cell death.^16^ In the working myocardium, cAMP can activate protein kinase A (PKA) and/or the exchange protein directly activated by cAMP (Epac) to mediate diverse biological effects, including cardiac remodeling and hypertrophy.^17, 18, 19, 20, 21, 22^ In addition to tmACs, cAMP can also be generated by soluble adenylyl cyclase (sAC), which is not regulated by heterotrimeric G proteins or forskolin (FSK), but can be activated by bicarbonate (HCO_3_^−^) and Ca^2+^.^16, 23, 24^ sAC was found inside mitochondria in the brain and liver and in certain mammalian cell types.^25, 26, 27, 28, 29^ In the liver and brain, in response to HCO_3_^−^ and/or Ca^2+^, mitochondrial cAMP stimulates oxidative phosphorylation and ATP production.^30^ In coronary endothelial cells, HCO_3_^−^ indirectly modulates the cell fate through apoptosis.^31, 32^ As a result, this pathway serves as a mechanism for metabolic adaptation to mitochondrial dysfunction and could be a potential novel target to treat genetic mitochondrial diseases.^33^ Altogether, these findings suggest that mitochondrial sAC functions as a metabolic sensor to stimulate mitochondrial biological functions. If proven in primary cardiomyocytes, this intramitochondrial cAMP pathway might have clinical implication in HF as patients diagnosed with HF have markedly impaired mitochondrial metabolism and cAMP signaling, both contributing to cardiomyocyte dysfunction.^16, 34^

Intrigued by these previous findings, we tested the existence of a cAMP mitochondrial pathway in differentiated adult and neonatal cardiomyocytes and observed that activation of this pathway prevents various cell deaths. Our results also show that cardiac mitochondria isolated from adult rat hearts contain a truncated form of sAC (sAC_t_) as a source of cAMP as well as Epac1. A role of this local pathway is to control mitochondrial Ca^2+^ entry through the MCU and to prevent the deleterious consequences of mitochondrial Ca^2+^ overload such as dissipation of mitochondrial membrane potential (ΔΨm) and induction of MPT. Interestingly, this mitochondrial sAC_t_-Epac1-MCU pathway remains functional in a rat model of HF induced by aortic stenosis and its activation prevents MPT.

## Results

### Mitochondrial cAMP prevents cardiac cell death, apoptosis as well as necrosis

**Figure 1:**
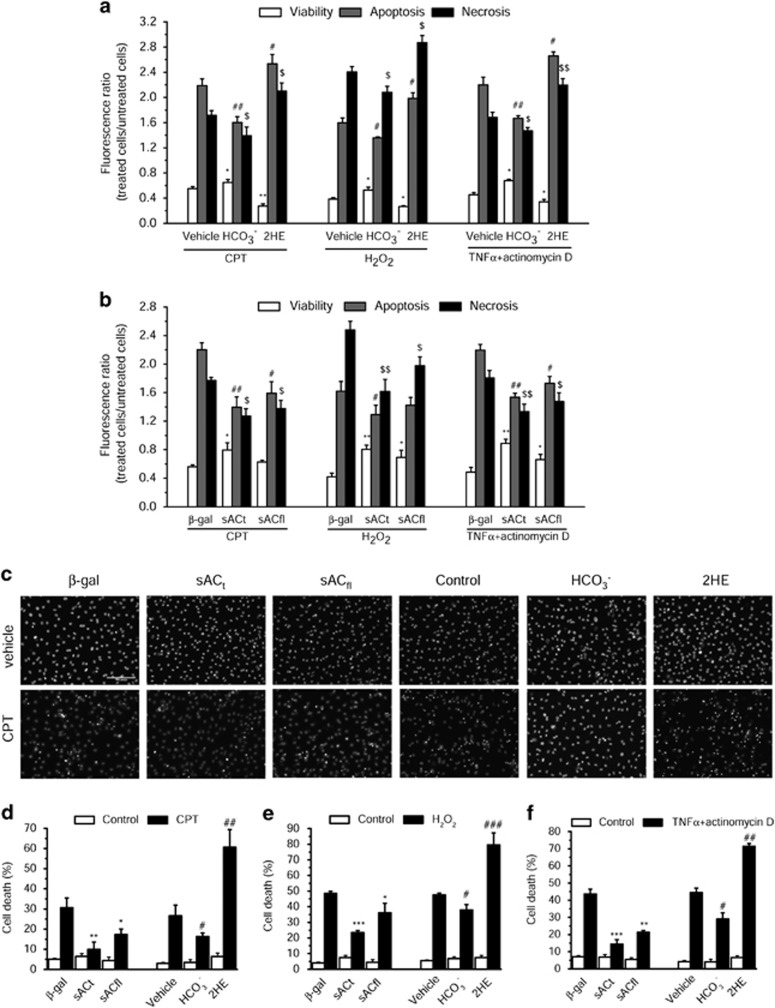
Mitochondrial cAMP protects cell death induced by camptothecin (CPT), hydrogen peroxide (H_2_O_2_) and tumor necrosis factor-*α* (TNF*α*) in neonatal cardiomyocytes. (**a**) Cells were treated with vehicle, 15 mM HCO_3_^−^ and 25 *μ*M 2HE in the presence of 10 *μ*M CPT for 48 h or 300 *μ*M H_2_O_2_ for 24 h or 10 ng/ml TNF*α*/0.1 *μ*g/ml actinomycin D for 24 h. (**b**) Cells were infected by adenoviruses encoding *β*-galactosidase (*β*-gal), sAC_t_ and sAC_fl_ for 24 h and then treated with 10 *μ*M CPT for 48 h or 300 *μ*M H_2_O_2_ for 24 h or 10 ng/ml TNF*α*/0.1 *μ*g/ml actinomycin D for 24 h. *P<0.05, **P<0.01 *versus* vehicle or *β*-gal viability; ^#^*P*<0.05, ^##^*P*<0.01 *versus* vehicle or *β*-gal apoptosis; ^$^*P*<0.05, ^$$^*P*<0.01 *versus* vehicle or *β*-gal necrosis (*n*=3). (**c**) Representative fluorescence images of nuclear staining with Hoechst 33342. (**d**–**f**) Quantitative analysis of cell death rate. Cells were infected with adenoviruses encoding *β*-gal, sAC_t_ and sAC_fl_ for 24 h and then treated with CPT (10 *μ*M) for 48 h, H_2_O_2_ (300* μ*M) or TNF*α*/actinomycin D (10 ng/ml, 0.1 *μ*g/ml) for 24 h, or cells were treated with vehicle, 15 mM HCO_3_^−^ and 25 *μ*M 2HE in the presence of CPT (10 *μ*M) for 48 h, H_2_O_2_ (300 *μ*M) or TNF*α*/actinomycin D (10 ng/ml, 0.1 *μ*g/ml) for 24 h. *P<0.05, **P<0.01, ***P<0.001 *versus β*-gal; ^#^*P*<0.05, ^##^*P*<0.01, ^###^*P*<0.001 *versus* vehicle (*n*=3)

To evaluate the capacity of sAC and cAMP to regulate the cardiomyocyte cell fate, we infected primary neonatal cardiomyocytes with two adenoviruses to overexpress the full-length sAC (sAC_fl_) and the sAC_t_, respectively, 24 h before cell death induction by three different cell death inducers, that is, camptothecin, H_2_O_2_ and TNF-*α*+actinomycin D. We showed that the stimulation of endogenous sAC with HCO_3_^−^ as well as overexpression of sAC_t_ prevented the various cell death modalities, apoptosis as well as necrosis measured by annexin/7-AAD labeling (Figures 1a and b). In contrast, inhibition of sAC with 2-hydroxyestradiol (2HE), a sAC inhibitor,^29^ aggravated significantly cell deaths (Figure 1a). We observed also that cAMP protects from nuclei alterations measured by counting Hoechst-stained nuclei exhibiting morphologic and biochemical alterations, that is, pycnosis and karryorrhexis (Figures 1c–f).

### Mitochondrial sAC produces locally cAMP and regulates ΔΨm upon calcium overload

**Figure 2:**
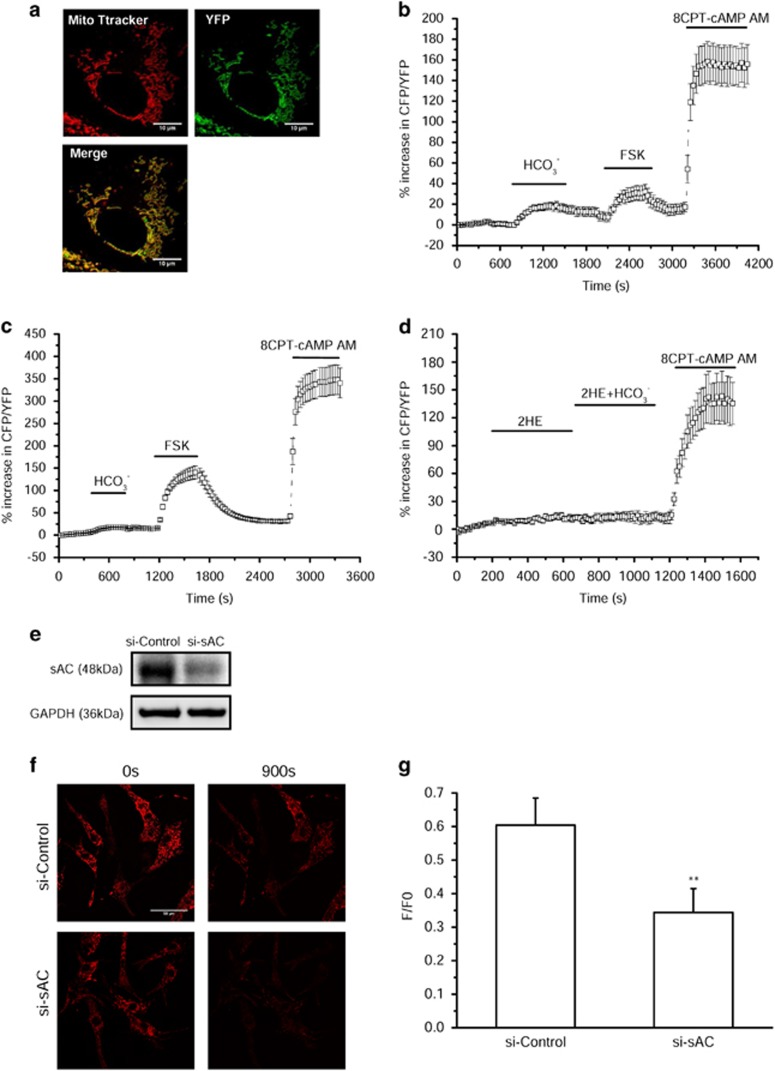
Mitochondrial sACt produces locally cAMP and regulates ΔΨm upon calcium overload. (**a**) Mitochondrial localization of the 4mt-Epac-S^H187^ cAMP sensor in rat isolated neonatal cardiomyocytes. Confocal images of cardiomyocytes infected with 4mt-Epac-S^H187^ (green) and stained with MitoTracker Red. The colocalization of 4mt-Epac-S^H187^ with MitoTracker is shown in yellow. Bar scale, 10 *μ*M. (**b** and **c**) Representative kinetics of percentage increase in CFP/YFP recorded in rat neonatal cardiomyocytes infected with either 4mt-Epac-S^H187^ (**b**) or Epac-S^H187^ sensor (**c**) and sequentially stimulated with 24 mM HCO_3_^−^, 25 *μ*M FSK and 20 *μ*M 8CPT-cAMP AM. (**d**) Representative kinetics of percentage increase in CFP/YFP recorded in rat neonatal cardiomyocytes infected with 4mt-Epac-S^H187^ exposed to 25 *μ*M 2HE in the absence or presence of 24 mM HCO_3_^−^, and finally to 20 *μ*M 8CPT-cAMP AM (**b**, *n*=19; **c**, *n*=6; **d**, *n*=7). (**e**) sAC expression in neonatal rat cardiomyocytes transfected with non-targeting small interfering RNA (siRNA) (si-Control) or sAC siRNA (si-sAC). (**f**) Representative confocal images of tetramethylrhodamine, methyl ester (TMRM)-labeled permeabilized neonatal rat cardiomyocytes transfected with si-Control or si-sAC at time 0 s (left) and 900 s (right) after Ca^2+^ (600 nM) addition. Bar scale, 50  *μ*M. (**g**) Averaged values of mitochondrial membrane potential (measured as F/F0, where F is the TMRM fluorescence signal at 900 s and F0 is the signal at time 0 s of Ca^2+^ addition) (*n*=50). **P<0.01 *versus* si-Control

As mitochondria may be impermeant to cytosolic cAMP,^35^ we constructed an adenovirus encoding a cAMP-sensitive fluorescence resonance energy transfer (FRET) sensor (Epac-S^H187^)^36^ fused with a 4mt sequence and infected rat neonatal cardiomyocytes with this sensor, 4mt-Epac-S^H187^. The localization of 4mt-Epac-S^H187^ in mitochondria was shown by colocalization of its green fluorescence with mitotracker red fluorescence (Pearson's coefficient: 0.92±0.02, *n*=6) (Figure 2a). Following infection with 4mt-Epac-S^H187^, we sequentially treated the cells with HCO_3_^−^ to activate sAC, FSK to activate tmAC and 8-CPT-2′-OMe-cAMP-AM (8CPT-cAMP AM), a permeant cAMP analog, to directly activate the sensor (Figure 2b). Addition of HCO_3_^−^ induced an increase in cAMP measured by 4mt-Epac-S^H187^ (Figure 2b), which was virtually absent when the cells were infected with the cytoplasmic cAMP sensor Epac-S^H187^ (Figure 2c). On the contrary, FSK induced a large response of cAMP measured with the cytoplasmic sensor (Figure 2c) and a smaller response of cAMP measured with the mitochondrial sensor (Figure 2b). These results are compatible with HCO_3_^−^-activating sAC in mitochondria and FSK increasing cAMP in the cytoplasm by activating tmAC. The small response to FSK observed with 4mt-Epac-S^H187^ might be due to incomplete targeting of the probe to mitochondria. Of note, 4mt-Epac-S^H187^ has a much higher dynamic range than previously published sensors such as mito-EpacH90,^35^ so that even a small expression of the probe in the cytosol would lead to a detectable signal. Alternatively, the small response to FSK measured with 4mt-Epac-S^H187^ might be due to Ca^2+^ stimulation of sAC in the matrix upon FSK stimulation as suggested previously,^29^ or to a small permeability of the mitochondrial inner membrane (IM) to cAMP. Interestingly, 2HE totally prevented the mitochondrial cAMP increase elicited by HCO_3_^−^ (Figure 2d). This confirms the involvement of a mitochondrial sAC as a source of cAMP in cardiomyocytes.

To address the role of sAC in the regulation of mitochondrial function, cardiomyocytes were transfected with siRNA control and siRNA against sAC for 48 h. Next, they were loaded with the fluorescent ΔΨm indicator, TMRM, permeabilized and treated with Ca^2+^. The decrease in the level of sAC did not induce any changes of the mitochondrial network (Figures 2e and f). However, the measure of the TMRM fluorescence ratio showed that silencing of sAC markedly aggravated the loss of ΔΨm induced by Ca^2+^, suggesting a role of cAMP in ΔΨm control in stress conditions.

### cAMP is produced by sACt in isolated mitochondria

**Figure 3:**
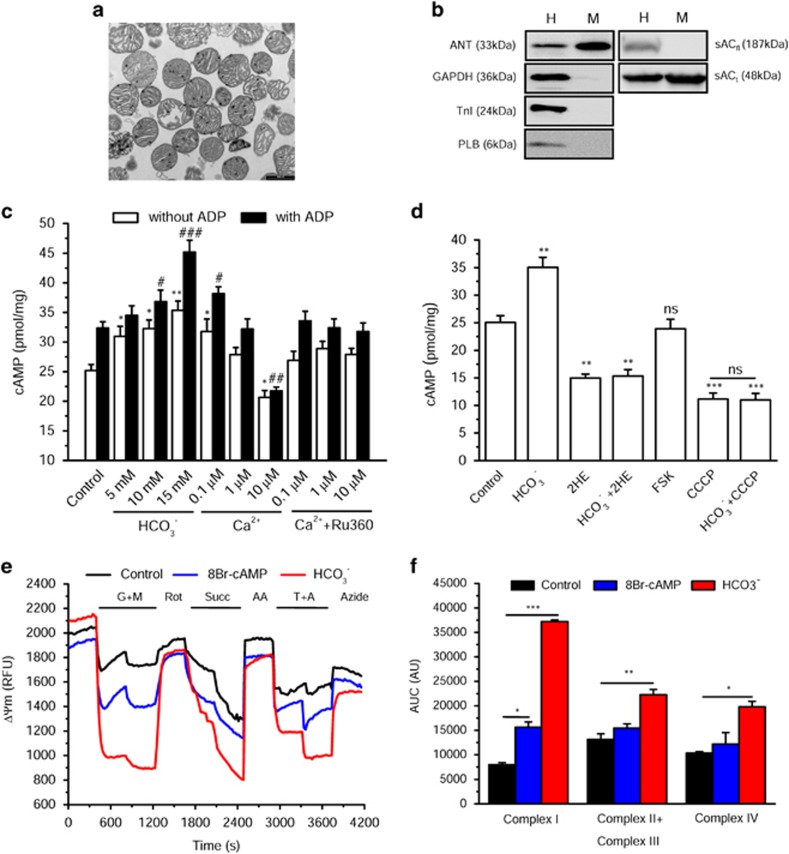
cAMP produced by sAC regulates mitochondrial transmembrane inner potential (ΔΨm). (**a**) Transmission electron microscopy image of isolated subsarcolemmal mitochondria from rat heart ventricles. Bar scale, 1 *μ*M. (**b**) Purity analysis of mitochondrial fraction by western blot. Protein ANT, inner membrane; glyceraldehyde 3-phosphate dehydrogenase (GAPDH), cytosol; PLB, sarcoplasmic reticulum; TnI; myofibrils; sAC_fl_ and sAC_t_ were probed in heart homogenate (H) and mitochondria (M). Results are representative of three independent experiments. (**c**) cAMP levels produced in isolated mitochondria in the presence of HCO_3_^−^, Ca^2+^ and Ca^2+^+Ru360 (a MCU inhibitor, 1 nM), under basal condition or upon stimulation with 1.65 mM ADP, determined by enzyme-linked immunosorbent assay (ELISA) (*n*=4–5). (**d**) cAMP levels in isolated mitochondria under basal condition or in the presence of 15 mM HCO_3_^−^, 25 *μ*M 2HE, HCO_3_^−^+2HE, 25 *μ*M FSK, 5 *μ*M CCCP or CCCP+HCO_3_^−^, determined by ELISA. Control, untreated mitochondria; NS, not significant (*n*=3–7). (**e**) ΔΨm was evaluated with Rhod123 fluorescence in the absence or presence of 1 mM 8Br-cAMP or 15 mM HCO_3_^−^ in isolated cardiac mitochondria with different respiratory substrates: 0.25 mM malate (M) and 0.5 mM glutamate (G) for complex I (inhibited by 2 *μ*M rotenone (Rot)); 0.5 mM succinate (Succ) for complexes II and III (blocked by the complex III inhibitor antimycin A (AA), 0.25 *μ*g/ml) and 0.05 mM TMPD (T) with 0.2 mM ascorbate (A) for complex IV (inhibited by 5 mM sodium azide). RFU, relative fluorescence unit. (**f**) Comparison of 8Br-cAMP and HCO_3_^−^ effects on ΔΨm stimulated with various respiratory substrates. Areas under the curve (AUC) were calculated from experiments such as that shown in (**e**) (*n*=3). AU, arbitrary units. *P<0.05, **P<0.01, ***P<0.001 *versus* Control; ^#^*P*<0.05, ^##^*P*<0.01 *versus* Control with ADP

Mitochondria were isolated from rat heart ventricles by differential centrifugation and extensive washes.^37^ First, we analyzed their morphology by transmission electron microscopy (Figure 3a) and their purity by western blotting (Figure 3b). As expected, mitochondria appeared round-shaped (mean diameter, 0.8 *μ*m) and presented numerous cristae, compatible with a high respiratory capacity. In comparison with rat ventricles homogenate (H), isolated mitochondria (M) were enriched in the adenine nucleotide translocase (ANT), an IM protein and almost not contaminated by cytosolic proteins such as GAPDH, myofibrillar proteins such as troponin I (TnI) and sarcoplasmic reticulum-associated proteins such as phospholamban (PLB) (Figure 3b). Using specific monoclonal antibodies, we detected the sAC_t_ (48 kDa) and the sAC_fl_ (187 kDa) in H fraction, whereas only the short form, which is the active form,^38^ was found in the mitochondria preparation (Figure 3b). Next, we measured cAMP production in freshly isolated mitochondria. We observed that HCO_3_^−^ and also, to a lesser extent, Ca^2+^-stimulated cAMP production in a dose-dependent manner and potentiated the response to ADP (Figure 3c). Although a small stimulatory effect of Ca^2+^ on cAMP production was observed at 0.1 *μ*M, when increasing the concentration to 10 *μ*M, mitochondria lost their membrane potential (not shown), lowering markedly cAMP production (Figure 3c). Ca^2+^ effects were abolished in the presence of RU360, confirming that the effect on cAMP levels is due to a specific uptake of Ca^2+^ within the matrix. Similarly, when mitochondria were depolarized by the protonophore carbonyl cyanide *m*-chlorophenyl hydrazone (CCCP), no cAMP production was detected even in the presence of HCO_3_^−^ (Figure 3d). Moreover, 2HE reduced basal and fully blocked HCO_3_^−^-stimulated cAMP production (Figure 3d). As a control, FSK had no stimulatory effect on cAMP in isolated mitochondria, confirming clearly the absence of tmAC within mitochondria (Figure 3d).

### cAMP increases ΔΨm, respiration and ATP levels

**Figure 4:**
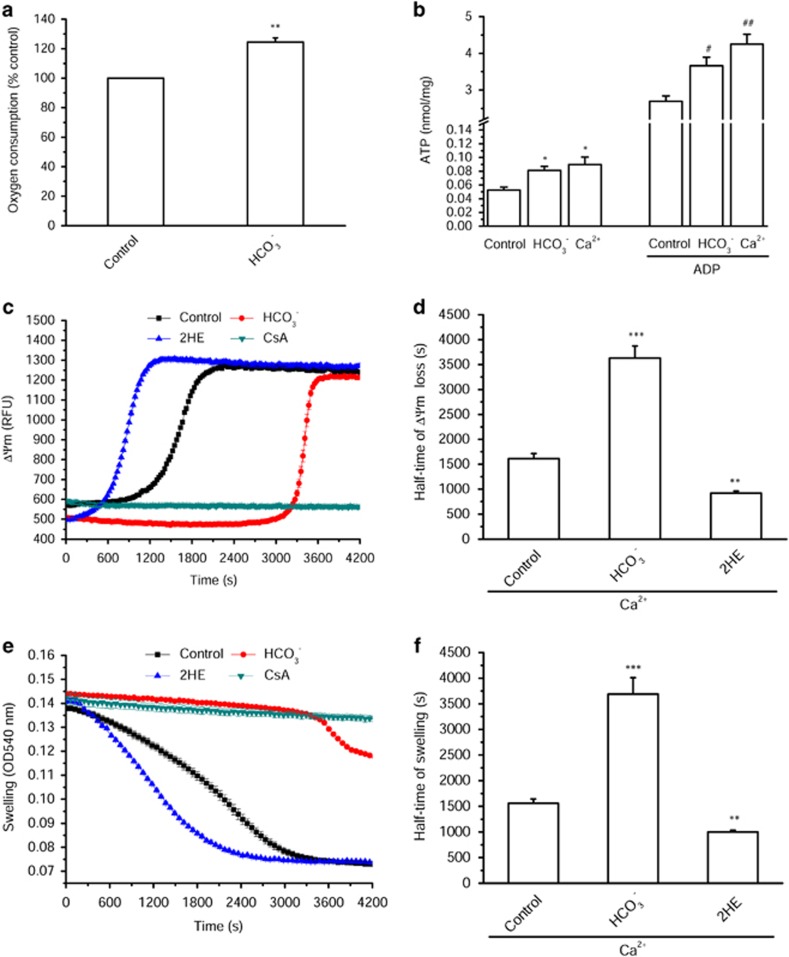
cAMP regulates mitochondrial respiration, ATP levels and Ca^2+^ induced mitochondrial depolarization and swelling. (**a**) Oxygen consumption of mitochondria measured with the probe MitoXpress in the presence or absence of 15 mM HCO_3_^−^ driven by 2.5 mM malate and 5 mM glutamate with 1.65 mM ADP. Control was normalized at 100% (*n*=5). (**b**) ATP production in the presence of 15 mM HCO_3_^−^ and 0.1 *μ*M Ca^2+^ with or without 1.65 mM ADP stimulation driven by 5 mM succinate (*n*=4). *P<0.05 *versus* Control; ^#^*P*<0.05, ^##^*P*<0.01 *versus* Control with ADP. (**c**) Effect of 15 mM HCO_3_^−^, 25 *μ*M 2HE and 5 *μ*M CsA on ΔΨm loss induced by 10 *μ*M Ca^2+^. (**d**) Average half-time values of ΔΨm loss induced by 10 *μ*M Ca^2+^ calculated from panels (**c**) (*n*=7–20). (**e**) Effect of 15 mM HCO_3_^−^, 25 *μ*M 2HE and 5 *μ*M CsA on mitochondrial swelling induced by 10 *μ*M Ca^2+^. (**f**) Average half-time values of mitochondrial swelling induced by 10 *μ*M Ca^2+^ calculated from panel **e** (*n*=7–20). **P<0.01, ***P<0.001 *versus* Control

Next, the ΔΨm was montored with the probe, rhodamine 123 (Rhod123), in the presence of various respiratory substrates (Figures 3e and f). We used 8Br-cAMP, a membrane-permeant cAMP analog, as a control, and HCO_3_^−^ to stimulate endogenous production of cAMP. Figure 3e shows that 8Br-cAMP slightly hyperpolarized mitochondria in condition of complex I-driven respiration, but failed to have any effect in the presence of respiratory substrates for complexes II–IV. In contrast, HCO_3_^−^ triggered a hyperpolarization in all conditions of substrates (Figure 3f). This hyperpolarization was accompanied by an increase in oxygen consumption in response to HCO_3_^−^ (Figure 4a). Finally, when mitochondria were stimulated by HCO_3_^−^ or Ca^2+^, this led to an increase in ATP production both in the absence and presence of ADP (Figure 4b). These data indicate that cAMP produced by a mitochondrial sAC stimulates the oxidative phosphorylation increasing ΔΨm and mitochondrial ATP synthesis.

### cAMP delays Ca^2+^-induced MPT

We hypothesized that the cyclic nucleotide could have a role in the regulation of MPT.^39^ In isolated cardiac mitochondria, MPT can be elicited by 10 *μ*M Ca^2+^ and prevented by 5 *μ*M CsA and detected as a loss of ΔΨm and a matrix swelling.^37^ We used two robust miniaturized assays^37, 40^ to concomitantly measure the effect of sAC inhibition by 25 *μ*M 2HE on mitochondrial depolarization (Figures 4c and d) and matrix swelling (Figures 4e and f) induced by 10 *μ*M Ca^2+^. sAC inhibition by 2HE accelerated the depolarization (Figures 4c and d) and swelling (Figures 4e and f) induced by Ca^2+^, as shown by the decreased half-time of ΔΨm loss and swelling (Figures 4d and f). Conversely, 15 mM HCO_3_^−^ slowed both processes (Figures 4d and f), suggesting that cAMP elevation confers a protection of mitochondria from Ca^2+^-induced MPT.

### Mitochondrial cAMP effects are independent of PKA

cAMP effects are classically mediated by activation of two main effectors, PKA and Epac to regulate a plethora of biological functions in the heart.^19^ In mitochondria, PKA has been reported to be associated with outer membrane (OM) or to be in the matrix for controlling mitochondrial dynamics and oxidative metabolism.^35^^,^^41^^,^^42^ We thus examined whether PKA was involved in the mitochondrial cAMP effects by testing the effects of two different pharmacological PKA inhibitors, H89 and KT5720, on the induction of MPT by Ca^2+^. As shown in Supplementary Figure 1, these inhibitors had no significant effect on ΔΨm and swelling, indicating that PKA may not be involved in MPT regulation.

### Epac1 mediates cAMP effect on respiration and MPT

**Figure 5:**
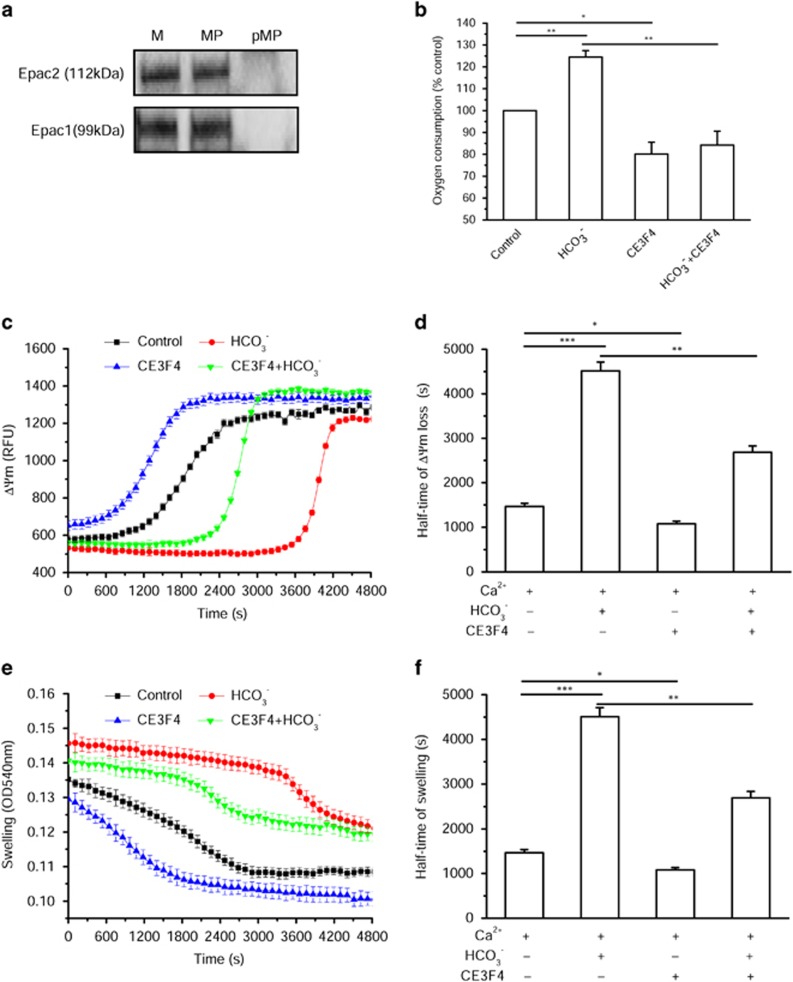
Epac1 mediates cAMP effect on respiration and permeability transition. (**a**) Western blot analysis of Epac1 and Epac2 isoforms in mitochondria (M), mitoplast (MP) and postmitoplast fraction (pMP). (**b**) Oxygen consumption measurement with the MitoXpress probe in the absence or presence of 15 mM HCO_3_^−^, 50 *μ*M CE3F4 and HCO_3_^−^+CE3F4. Control, untreated mitochondria, has been normalized to 100% (*n*=5). (**c**) Effects of CE3F4 on ΔΨm induced by 10 *μ*M Ca^2+^. (**d**) Average half-time values of ΔΨm loss induced by 10 *μ*M Ca^2+^ calculated from experiments such as that shown in (**c**) (*n*=15). (**e**) Effects of CE3F4 on mitochondrial swelling induced by 10 *μ*M Ca^2+^. (**f**) Average half-time values of mitochondrial swelling induced by 10 *μ*M Ca^2+^ calculated from experiments such as that shown in (**e**) (*n*=15). *P<0.05, **P<0.01, ***P<0.001 *versus* Control

Then, we checked the expression of Epac isoforms. As shown in Figure 5a, both Epac1 and Epac2 isoforms were found in isolated cardiac mitochondria as well as in mitoplasts generated by osmotic shock, but were absent in the postmitoplast supernatant. This suggests that Epac can be anchored to the IM facing the mitochondrial matrix or the intermembrane space or localized in the matrix.

To evaluate the functional role of Epac, we used three pharmacological Epac inhibitors exhibiting different specificities and tested their effects on Ca^2+^-induced depolarization and swelling as well as oxygen consumption. We used ESI09, a pan-Epac inhibitor, ESI05, an Epac2-selective inhibitor,^21, 43^ and CE3F4, an Epac1-selective inhibitor.^44^ As shown in Figure 5b, Epac1 inhibition with 50 *μ*M CE3F4 decreased basal oxygen consumption and also prevented the stimulatory effect of HCO_3_^−^. Moreover, CE3F4 accelerated Ca^2+^-induced depolarization (Figures 5c and d) and swelling (Figures 5e and f). Similar findings were obtained with ESI09 but not with ESI05 (Supplementary Figure 2). These data thus point to Epac1 as a key effector in mitochondrial cAMP effects.

**Figure 6:**
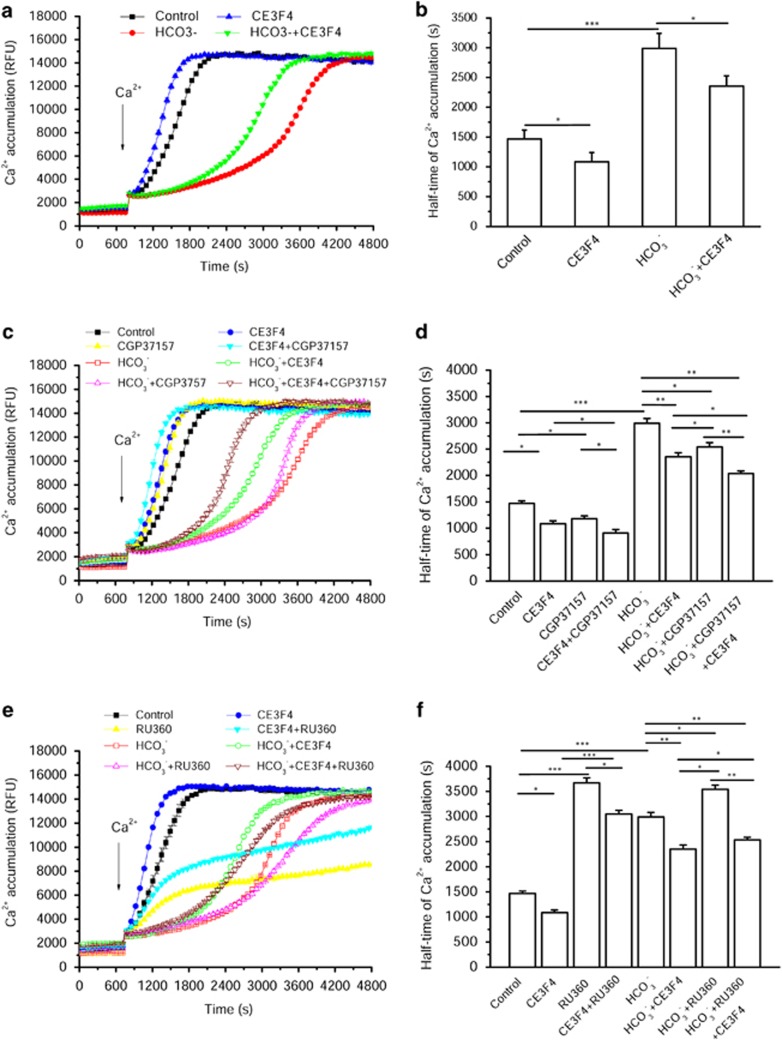
Epac1 prevents Ca^2+^ entry into mitochondria via the Ca^2+^ uniporter and not the Na^+^/Ca^2+^ exchanger. (**a**) Measurement of Ca^2+^ accumulation in isolated mitochondria using Rhod-2. HCO_3_^−^ was used at 15 mM, and CE3F4 was used at 50 *μ*M. (**b**) Half-time of Ca^2+^ entry into mitochondria calculated from experiments such as that shown in (**a**) (*n*=5). (**c**) Time course of Ca^2+^ accumulation in isolated mitochondria in the presence of 15 mM HCO_3_^−^, 50 *μ*M CE3F4 and 10 *μ*M CGP37157 (a mNCX inhibitor). (**d**) Half-time of Ca^2+^ accumulation into mitochondria calculated from experiments such as that shown in (**c**) (*n*=5). (**e**) Time course of Ca^2+^ accumulation in isolated mitochondria in the presence of 15 mM HCO_3_^−^, 50 *μ*M CE3F4 and 0.4 nM Ru360 (a MCU inhibitor). (**f**) Half-time of Ca^2+^ accumulation into mitochondria calculated from experiments such as that shown in (**e**) (*n*=5). *P<0.05, **P<0.01 and ***P<0.001

Next, we tested the effect of Epac1 on the level of matrix Ca^2+^ using the Rhod-2 probe, and CGP37157, a mitochondrial Na^+^/Ca^2+^ exchanger (mNCX) inhibitor, appeared to accelerate Ca^2+^ entry in isolated mitochondria (Figures 6c and d). This effect was similar to that of CE3F4. However, the combination of both inhibitors produced an additive effect, suggesting that they act via two distinct mechanisms. Thus, it is unlikely that Epac1 regulates mNCX. To examine the role of MCU, we used RU360, a highly specific MCU inhibitor. As anticipated, RU360 (from 0.2 to 1 nM) induced a dose-dependent inhibition of Ca^2+^ entry (Supplementary Figure 3). Interestingly, inhibition of Epac in the presence of non-maximal concentrations of RU360 partially restored Ca^2+^ entry within mitochondria (Figures 6e and f and Supplementary Figure 3), but this effect was abrogated when the MCU was fully inhibited with 1 nM RU360 (Supplementary Figure 3a). These results suggest that MCU is the major effector of Epac1 for the regulation of mitochondrial Ca^2+^ movements.

### Epac1 mediates mitochondrial Ca^2+^accumulation and ΔΨm loss in cardiomyocytes

**Figure 7:**
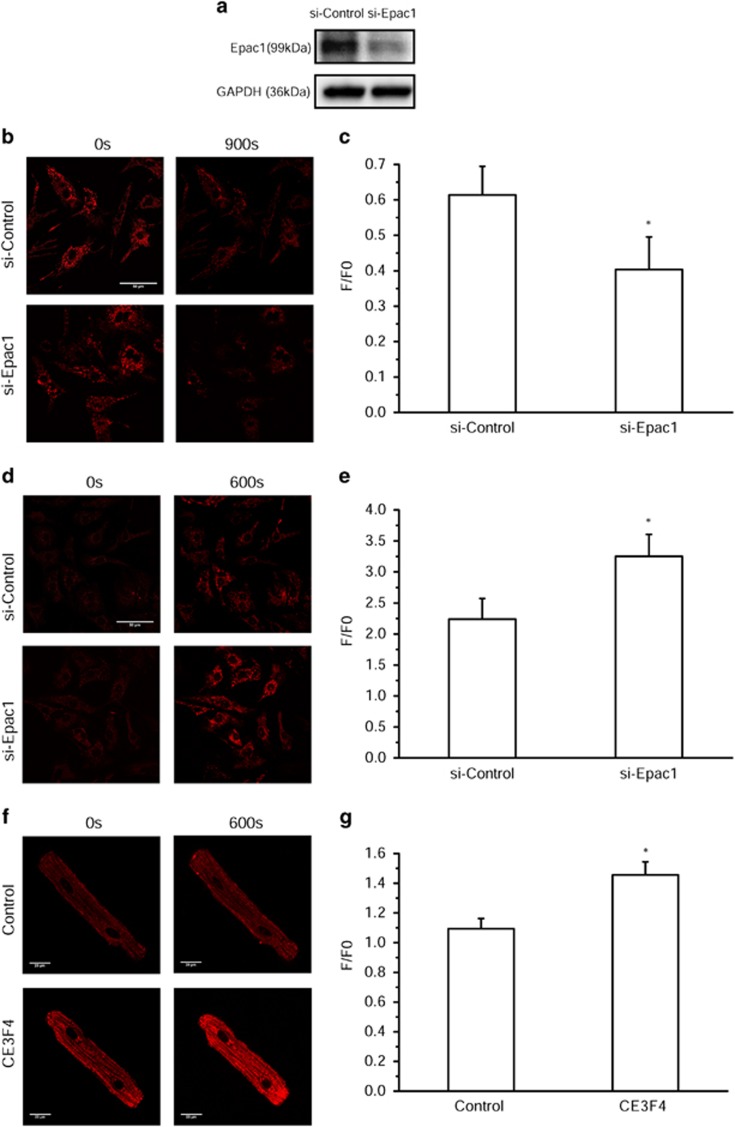
Epac1 mediates mitochondrial calcium accumulation and ΔΨm loss *in cellulo*. (**a**) Epac1 expression in neonatal rat cardiomyocytes transfected with non-targeting small interfering RNA (siRNA) (si-Control) or Epac1 siRNA (si-Epac1). (**b**) Representative confocal images of TMRM-labeled permeabilized neonatal rat cardiomyocytes transfected with si-Control or si-Epac1 at time 0 s (left) and 900 s (right) after Ca^2+^ (600 nM) addition. Bar scale, 50 *μ*M. (**c**) Averaged values of ΔΨm (measured as F/F0, where F is the TMRM fluorescence signal at 900 s and F0 is the signal at time 0 s of Ca^2+^ addition) (*n*=36). (**d**) Representative confocal images of Rhod-2 AM-labeled permeabilized neonatal rat cardiomyocytes transfected with si-Control or si-Epac1 at time 0 s (left) and 600 s (right) after Ca^2+^ (200 nM) addition. Bar scale, 50 *μ*M. (**e**) Averaged values of intramitochondrial Ca^2+^ accumulation (measured as F/F0, where F is the Rhod-2 fluorescence signal at 600 s and F0 is the signal at time 0 s of Ca^2+^ addition) (*n*=30). (**f**) Representative confocal images of Rhod-2 AM-labeled permeabilized adult rat ventricular myocytes at time 0 s (left) and 600 s (right) after Ca^2+^ (200 nM) addition in the absence (top) or presence (bottom) of CE3F4. Bar scale, 20 *μ*M. (**g**) Averaged values of intramitochondrial Ca^2+^ accumulation (measured as F/F0, where F is the Rhod-2 fluorescence signal at 600 s and F0 is the signal at time 0 s of Ca^2+^ addition) (*n*=10). *P<0.05 *versus* si-Control or Control

The Epac1 silencing by siRNA indicated that a decreased level of Epac1 in neonatal rats decreased ΔΨm (Figures 7a and c) and in parallel accelarated the mitochondrial calcium entry (Figures 7d and e), as does the inhibitor CE3F4 in adult permeabilized cardiomyocytes upon addition of Ca^2+^ (Figures 7f and g). At this concentration, Ca^2+^ did not affect ΔΨm, avoiding any artifact since most ions and metabolites transports are dependent of the ΔΨm (Supplementary Figures 4a and b). We also checked that our conditions of fluorescence excitation did not trigger MPT (Supplementary Figures 4c and d). Altogether, these results suggest that Epac1 has a role in reducing the entry of Ca^2+^ in mitochondria, and then indirectly stabilizes the ΔΨm in primary cardiomyocytes.

### The mitochondrial cAMP pathway can prevent MPT in HF rat model

**Figure 8:**
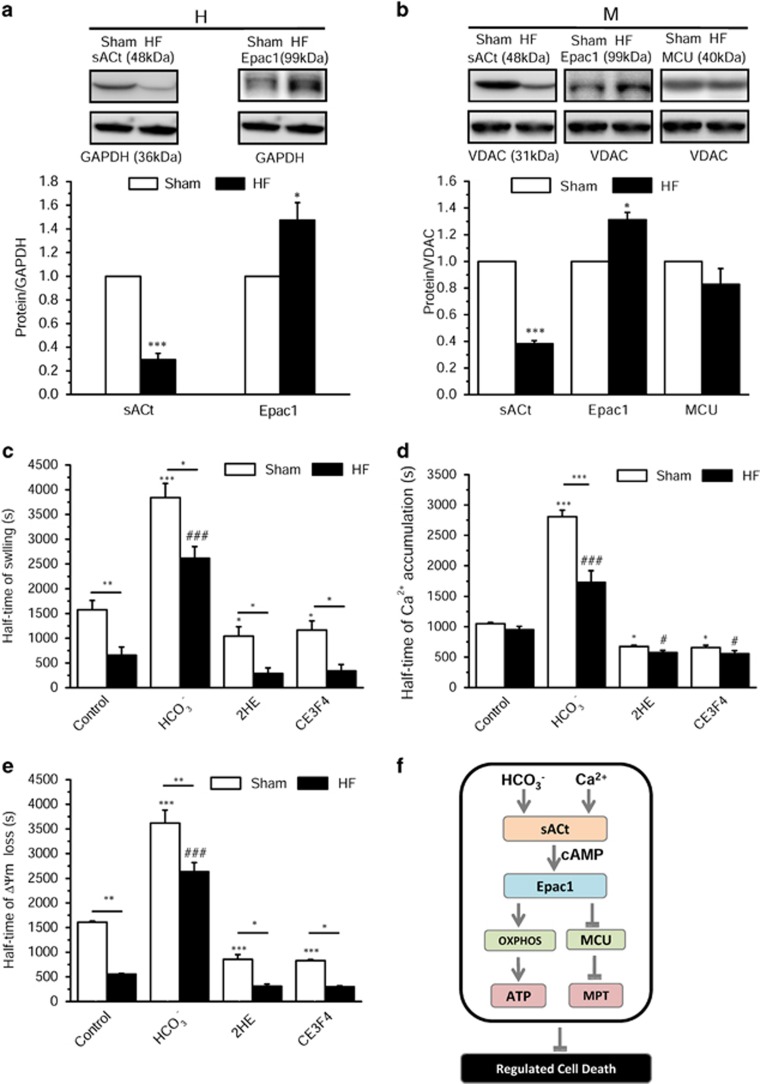
Expression levels of sACt, Epac1 and MCU in hearts and mitochondria isolated from sham and HF rats and cAMP regulation of ΔΨm and Ca^2+^ uptake. (**a**) Expression level of sAC and Epac1 in sham (white bars) and HF (black bars) heart homogenates (H) normalized by glyceraldehyde 3-phosphate dehydrogenase (GAPDH). Representative western blot images are shown on top (sham on left and HF on right). (**b**) Expression level of sAC_t_, Epac1 and MCU proteins relative to voltage-dependent anion channel (VDAC) in isolated mitochondria (M) in HF *versus* sham. Representative blots are shown on top (sham on left and HF on right). Data are mean±S.E.M. of four sham and four HF rats, detected in four independent immunoblots. (**c**) Half-time of ΔΨm loss induced by 10 *μ*M Ca^2+^ calculated from experiments such as shown in Supplementary Figures 6a and b. (**d**) Half-time of Ca^2+^ accumulation calculated from experiments such as shown in Supplementary Figures 6c and d. (**e**) Half-time of swelling induced by 10*μ*M Ca^2+^ calculated from experiments such as that shown in Supplementary Figures 6e and f. *P<0.05, **P<0.01, ***P<0.001 *versus* sham control; ^#^*P*<0.05, ^###^*P*<0.001 *versus* HF control (*n*=4). (**f**) Hypothetical scheme showing the local role of mitochondrial cAMP signaling pathway. Within the mitochondrion, HCO_3_^−^ and calcium stimulate the production of cAMP by sAC_t_, which activates mitochondrial cAMP production. In turn, cAMP stimulates oxidative phosphorylation and inhibits permeability transition via activation of mitochondrial Epac1

To evaluate the ability of the mitochondrial cAMP pathway to regulate MPT in a pathological model, we induced HF in rats by transverse aortic constriction (TAC) during 22 weeks.^45^ As shown in Supplementary Figure 5a, TAC rats showed a strong cardiac and lung hypertrophy. Accordingly, cardiac function and the fractional shortening of the left ventrice were diminished (Supplementary Figures 5b and c). Expression level of various proteins was analyzed in heart ventricle homogenates and mitochondrial fraction. As shown in Figures 8a and b, sAC_t_ protein expression was reduced and Epac1 expression was increased in homogenate and mitochondria from HF as compared with sham hearts. MCU expression was similar in mitochondrial fraction from HF and sham rats. To explore how HF affects mitochondrial Ca^2+^-induced MPT, Ca^2+^-induced mitochondrial depolarization and Ca^2+^ accumulation was measured in isolated mitochondria from HF and sham rats. As shown in Figures 8c and d and Supplementary Figures 6a–d, Ca^2+^ induced a faster depolarization and Ca^2+^ uptake in HF than in sham mitochondria. In line with this, Ca^2+^ induced a faster mitochondrial swelling in HF than in sham mitochondria (Figure 8e and Supplementary Figures 6e and f). This confirms that MPT is altered in HF, which could make mitochondria more vulnerable to Ca^2+^ overload.^46^ Interestingly, mitochondria from HF rats still responded to HCO_3_^−^ stimulation of mitochondrial cAMP production by sAC, by delaying ΔΨm loss, Ca^2+^ entry and MPT (i.e. matrix swelling). These effects were blunted by sAC or Epac1 inhibition with CE3F4 (Figures 8c–e and Supplementary Figures 6a–f).

## Discussion

In this study, we characterized a functional cAMP pathway within the mitochondria of neonatal and adult cardiomyocytes, which can regulate mitochondrial function and cell death. cAMP is locally produced within the mitochondria by a Ca^2+^/HCO_3_^−^-sensitive sAC_t_ and activates Epac1 to stimulate oxidative metabolism while preventing MPT by limiting mitochondrial Ca^2+^ accumulation via MCU. As HCO_3_^−^ production can be catalyzed by carbonic anhydrase from CO_2_ and H_2_O, CO_2_ being produced by the Krebs cycle and the pyruvate deshydrogenase inside mitochondrial matrix, our data thus link, for the first time, mitochondrial metabolism, cAMP and cell death in the heart, independently of cytosolic cAMP signaling.

Our data are in good agreement with pioneer studies revealing the existence of a mitochondrial cAMP signaling in various cell types.^27, 28, 29^ Prompted by the observation that a G-protein- and FSK-insensitive sAC is present in various organelles,^38, 47, 48, 49^ Acin-Perez *et al.*^27^ discovered a CO_2_-HCO_3_^−^-sAC-cAMP-PKA (mito-sAC) signaling cascade entirely contained within the mitochondria. This mito-sAC cascade serves as a metabolic sensor modulating ATP generation and ROS production in response to nutrient availability.^29^ By targeting the recently developed Epac-S^H187^ cAMP FRET sensor^36^ to the mitochondria, we showed that sAC activation by HCO_3_^−^ increases mitochondrial cAMP in neonatal cardiomyocytes, as shown earlier in HeLa and CHO cells.^35^ We showed that the constitutive mitochondrial cAMP signaling pathway regulates ΔΨm and MPT not only in healthy but also in failing heart mitochondria and that these functions are mediated by Epac1.

### A functional mito-sAC pathway in mitochondria from adult heart

Although it was already known that sAC can be localized into mitochondria,^47, 48, 49^ little was known about their biological function in the organelle. Here, we identified endogenous sAC_t_ in cardiac mitochondria and mitoplasts. We showed for the first time that increasing intramitochondrial cAMP level delays the onset of MPT, while stimulating oxygen consumption. Although HCO_3_^−^ and Ca^2+^ enhanced cAMP production, HCO_3_^−^ was more potent than Ca^2+^, which is in line with the fact that HCO_3_^−^ and Ca^2+^ stimulatory effects are not redundant: HCO_3_^−^ modulates the active site of sAC, whereas Ca^2+^ increases ATP affinity.^27^ Interestingly, a specific inhibitor of sAC, 2HE, totally prevented the effects of HCO_3_^−^ and Ca^2+^, indicating that sAC may be the unique source of mitochondrial cAMP.

### Effectors of mitochondrial cAMP

While PKA is the canonical mediator of cAMP in a number of cell functions and cell subcompartments, and was shown earlier to regulate mitochondrial ATP and ROS production,^27, 29, 50, 51^ PKA was clearly not involved in the induction of MPT by Ca^2+^ as H89 and KT5720 failed to modulate it. We thus focused our interest on Epac, because it emerged in the past decade as another important player in cAMP signaling.^20^ Although Epac possesses a mitochondrial-targeting sequence at its N terminus and has been shown to be localized inside mitochondria by heterologous expression,^52^ to our knowledge there has been no report on a role for this protein in mitochondrial function. Although the Epac2-selective inhibitor ESI05 had no effect, the non-selective inhibitor ESI09 or the Epac1-selective inhibitor CE3F4 antagonized the induction of MPT by Ca^2+^. This indicates that Epac1 but not Epac2 is involved in the regulation of MPT. We found also that CE3F4 inhibits oxygen consumption. As the efficiency of CE3F4 to regulate oxygen consumption with a better efficiency than MPT, we speculate that Epac1 could have several targets, which remain to be identified, regulating differentially various mitochondrial functions.

In neonatal rat cardiomyoctes, silencing of Epac1 modulated the Ca^2+^ entry and the ΔΨm. In the heart, Epac1 was recently shown to be localized and functionally involved also in nuclear signaling, whereas Epac2 is located at the T tubules and regulates arrhythmogenic sarcoplasmic reticulum Ca^2+^ leak.^53^ While the intermediate downstream effector(s) of mitochondrial Epac1 still need to be identified, our results indicate that Epac1 activation may inhibit MCU activity. This hypothesis is supported by the fact that inhibition of MCU, but not of mNCX, mimics the effects of mitochondrial cAMP elevation in preventing MPT. Thus, we propose that activation of mitochondrial Epac1 protects the organelle from Ca^2+^ overload and from subsequent MPT via MCU modulation.

### Possible implications of the mitochondrial cAMP pathway for cell death and cardioprotection

Ca^2+^ overload is considered as a conserved inducer of regulated cell death modalities.^54^ Using modulation of sAC by genetic and pharmacological manipulations in primary cardiomyocytes, our study demonstrates for the first time that activation of the mitochondrial cAMP pathway exerts an inhibition on MPT *in vitro* and on various cell death modalities, that is, extrinsic and intrinsic apoptosis as well as necrosis. Conversely, pharmacological inhibition of sAC increased markedly nuclear damage and cell death. Thus, the targeted activation of this mitochondrial cAMP pathway may preserve cardiomyocytes from mitochondrial Ca^2+^ overload and cell death *in vivo*. In that respect, in a pathological rat model of HF induced by pressure overload, which goes along with strong cardiac hypertrophy, cardiac function alteration, tissue remodeling, bioenergetic alterations and cardiomyocyte cell death,^45, 46^ sACt is downregulated and Epac1 is upregulated in mitochondria. However, the increase in Epac1 did not compensate the decrease of sACt in terms of function, suggesting that the level of cAMP is limiting for Epac1 in the control of MPT in cardiac mitochondria. Moreover, we found that the MPT alterations can be alleviated by stimulation of the mitochondrial cAMP pathway. Thus, this new mitochondrial cAMP/sAC_t_/Epac1/MCU pathway might have therapeutic implications to regulate cell death in cardiac pathologies, such as HF and/or myocardial infarction.^54, 55^

## Material and Methods

Unless specified, all reagents and chemicals are from Sigma-Aldrich (Saint-Quentin Fallavier, France) and of analytical grade.

### Animals

All animal care and experimental procedures conformed to the European Community guiding principles in the care and use of animals (Directive 2010/63/EU of the European Parliament) and authorizations to perform animal experiments according to this decree were obtained from the French Ministry of Agriculture, Fisheries and Food (No. D-92-283, 13 December 2012). All studies involving rats are reported in accordance with the ARRIVE guidelines for reporting experiments involving animals.^56^ A total of 60 healthy, 4 sham and 4 HF rats were used in the experiments described here.

### Surgical procedure and echocardiography

Male Wistar rats at 3 weeks of age (60–70 g; Janvier, Le Genest St Isle, France) were anesthetized with pentobarbital (60 mg/kg). The thoracic cage was opened and a stainless-steel hemoclip was placed on the ascending aorta, to promote HF after 22 weeks, as described previously.^57^ Sham-operated animals were used as controls. Cardiac structure and function was evaluated by echocardiograph. Cardiac and pulmonary hypertrophy was determined as a ratio of organ weight to tibia length and to body weight.^57^ Transthoracic two-dimensional-guided M-mode echocardiography of rats was performed using an echocardiograph with a 15 MHz linear transducer (Vivid 9; General Electric Healthcare, Vélizy Villacoublay, France) under 3% isoflurane gas anesthesia and fractional release was calculated as described.^57^

### Isolation of cardiac mitochondria

Mitochondria were isolated from the heart of adult male Wistar rats at 8–10 weeks of age (275–375 g; Janvier) as described.^37^ Briefly, the heart was rapidly removed and placed into a cold buffer containing 0.3 M sucrose, 0.2 mM EGTA and 5 mM TES (pH 7.2). The heart was grinded with Polytron fastly and homogenized by using the Potter. The homogenate was centrifuged at 500 × *g* for 10 min at 4 °C. Then, the supernatant was carefully removed and centrifuged again at 3000 × *g* for 10 min at 4 °C. The pellets were washed in the isolation buffer and the mitochondria were kept on ice until use within 3 h.

### Mitochondrial transmembrane potential and swelling in isolated mitochondria

Isolated mitochondria (25 *μ*g proteins) were incubated with Ca^2+^ and drugs in 96-well microtiter plates.^37^ ΔΨm was measured using the fluorescent probe, Rhod123 (excitation=485 nm and emission=535 nm; Enzo Life Sciences, Villeurbanne, France) in a buffer containing 200 mM sucrose, 10 mM MOPS, 10 *μ*M EGTA, 1 mM H_3_PO_4_, 5 mM succinate and 2 *μ*M rotenone (pH 7.4) using Tecan Infinite 200 spectrofluorimeter (Tecan, Männedorf, Switzerland). In parallel, matrix swelling was measured via light absorbance at 540 nm.^37^

### Oxygen consumption

Isolated mitochondria (50 *μ*g proteins) were incubated with drugs in a buffer containing 250 mM sucrose, 30 mM K_2_HPO_4_, 1 mM EGTA, 5 mM MgCl_2_, 15 mM KCl and 1 mg/ml bovine serum albumin (BSA) (pH 7.4) supplemented with respiratory substrates and MitoXpress, an oxygen-sensitive phosphorescent dye (LUXCEL, Cork, Ireland). Oxygen consumption was measured in real time for 60 min at 30 °C in 96-well plates using Tecan Infinite 200 (excitation=380 nm and emission=650 nm) in the presence of 1.65 mM ADP and with 5 mM malate and 12.5 mM glutamate.^37^

### Mitochondrial Ca^2+^ uptake in isolated mitochondria

Isolated mitochondria (25 *μ*g proteins) were incubated with 5 *μ*M Rhod-2 (Enzo Life Sciences) in the buffer containing 200 mM sucrose, 10 mM MOPS, 10 *μ*M EGTA, 1 mM H_3_PO_4_, 5 mM succinate and 2 *μ*M rotenone for 30 min in dark at room temperature. Afterwards, the mitochondria were washed two times. Then, the mitochondria were treated with various drugs for 10 min before applying Ca^2+^. Fluorescence was measured in real time for 60 min at room temperature in 96-well plates using Tecan Infinite 200 (excitation=552 nm and emission=581 nm).

### cAMP measurements by ELISA

cAMP measurements were performed according to the manufacturer's instructions using monoclonal anti-cAMP antibody-based direct cAMP ELISA Kit (New East Biosciences, King of Prussia, PA, USA) on freshly isolated mitochondria from rat hearts (500 *μ*g proteins per sample) treated or not by HCO_3_^−^, Ca^2+^ and Ca^2+^+Ru360 for 20 min at room temperature before centrifugation and lysis. The sensitivity of cAMP detection is 29.6 fmol/ml.^57, 58^

### ATP measurements

ATP measurements in isolated mitochondria were performed according to manufacturer's instructions using ATP Bioluminescence Assay Kit CLSII (Roche, Basel, Switzerland).

### Western blotting

Total mitochondrial proteins were resolved on 4–15% Tris-glycine SDS-PAGE gels and electroblotted onto polyvinylidene fluoride membranes (Bio-Rad, Marnes La Coquette, France). Following electrotransfer, membranes were blocked for 1 h at room temperature in 5% BSA-PBST (10 mM Tris-HCl, pH 8.0/150 mM NaCl/0.1% Tween-20). Next, membranes were incubated overnight at 4 °C with primary antibody. The day after, the membranes were washed six times with PBST and incubated with peroxidase-conjugated secondary antibody at room temperature for 1 h. Peroxidase activity was detected with enhanced chemiluminescence (ECL Advance Western Blotting Detection Kit; Thermo Scientific, Villebon sur Yvette, France). For protein detection, the following antibodies were used: sAC (Abcam Cambridge, UK; CEP Biotech, Tamarac, FL, USA), Epac1, 2 (Cell Signaling, Danvers, MA, USA), ANT (Abcam), GAPDH (Cell Signaling), VDAC (Genosphere, Paris, France), TnI (Cell Signaling), PLB (Cell Signaling) and MCU (Biorbyt, Berkeley, CA, USA).

### Construction of mitochondria-targeted FRET sensor for cAMP

The mitochondrial-targeting sequence 4mt, encoding four copies of the signal sequence from subunit VIII of human cytochrome *C* oxidase, was amplified using the Advantage Polymerase (Clontech, Mountain View, CA, USA) and primers F, 5′-ACTATAGGGAGACCCAAGCTTATG-3′ and R, 5′-TGGTGGCGGCAAGCTTCTTGCTCACCATGGTGGC-3′. The pcDNA-4mt-D3-cpv vector used as a matrix for amplification of 4mt was a kind gift from Dr. Roger Tsien (HHMI investigator at the University of California San Diego, San Diego, CA, USA). The PCR fragment was cloned into the *Hind*III restriction site of pcDNA3-Epac-S^H187^ using the Infusion HD Cloning System (Clontech). Epac-S^H187^ encodes for a fourth-generation Epac1-based cAMP sensor and was a kind gift from Dr. Kees Jalink (The Netherlands Cancer Institute, Amsterdam, Netherlands).^36^ Once the pcDNA-4mt-Epac-S^H187^ vector was amplified in Stellar *Escherichia coli* (Clontech) bacteria, its identity with parental sequences was verified by PCR using primers F, 5′-ACTCACTATAGGGAGACC-3′ and R, 5′-TGCGGCCGCCATGGTGGC-3′, and DNA double-strand sequencing (INSERM U1056 – UMR 5165 CNRS UPS – UDEAR, Toulouse, France). Adenoviruses encoding Epac-S^H187^ and 4mt-Epac-S^H187^ were generated by Welgen Inc (Worcester, MA, USA).

### Cardiomyocyte isolation, adenoviral infection and cell death evaluation

Adult and neonatal cardiomyocytes were isolated as described previously.^59,^^60^ For FRET experiments, neonatal cardiomyocytes were plated on 35-mm, laminin-coated culture dishes (10 *μ*g/ml) at a density of 4 × 10^5^ cells per dish. The day after, cells were infected with Epac-S^H187^ and 4mt-Epac-S^H187^ adenoviruses in Opti-MEM (Life Technologies, St Aubin, France) for 48 h. Similarly, adenoviruses expressing sACt and sACfl were used (generous gift from Pr. M Conti, University of California, San Francisco, CA, USA). For confocal microscopy experiments, adult cardiomyocytes were plated on 35-mm, laminin-coated culture dishes (10 *μ*g/ml) at a density of 2 × 10^4^ cells per dish. For cell death evaluation, neonatal cells were stained with Apoptosis/Necrosis Detection Kit (Abcam) for 1 h at room temperature as described by the manufacturer.

### siRNA transfection to knockdown sAC and Epac1

On-Target plus SMART pool siRNA, a mixture of four siRNA provided as a single reagent were purchased from Dharmacon (Lafayette, CO, USA). At day 0, neonatal cardiomyocytes were plated overnight on 35-mm, laminin-coated culture dishes (10 *μ*g/ml) at 4 × 10^5^. At day 1, the cells were transfected with 50 nM sAC/Epac1 or non-targeting control siRNA using Lipofectamine RNAi MAX Transfection Reagent (ThermoScientific, Waltham, MA, USA) for 48 h.

### Mitochondrial transmembrane potential measurement in neonatal cardiomyocytes

Isolated rat cardiomyocytes were loaded with 100 nM TMRM at 37 °C for 15 min. Afterwards, the sarcolemmal membrane was permeabilized by perfusion of digitonin (5 *μ*g/ml) in a Ca^2+^-free internal solution that contained 50 mM KCl, 80 mM potassium aspartate, 4 mM sodium pyruvate, 20 mM HEPES, 3 mM MgCl_2_, 3 mM Na_2_ATP, 5.8 mM glucose and 0.5 mM EGTA (pH 7.3 with KOH). Then, the free Ca^2+^ concentration in the internal solution was increased to 200 nM. The Ca^2+^ was calculated using the Maxchelator program from the Stanford University (Stanford, CA, USA). Images were acquired with a Leica (SP5) confocal microscope (Mannheim, Germany). Excitation was achieved by a white light laser fitted at 549 nm and emission collected at 570 nm. Analyses were made with Image J program (Wayne Rasband, National Institutes of Health, USA).

### Measurement of mitochondrial Ca^2+^ in cardiomyocytes

Isolated neonatal or adult rat cardiomyocytes were loaded with 5 *μ*M Rhod-2 at 37 °C for 30 min. To remove cytosolic Rhod-2, the sarcolemmal membrane was permeabilized by perfusion of digitonin (5 *μ*g/ml) in a Ca^2+^-free internal solution that contained 50 mM KCl, 80 mM potassium aspartate, 4 mM sodium pyruvate, 20 mM HEPES, 3 mM MgCl_2_, 3 mM Na_2_ATP, 5.8 mM glucose and 0.5 mM EGTA (pH 7.3 with KOH). After the sarcolemmal membrane was permeabilized, the free Ca^2+^ concentration in the internal solution was increased to 200 nM. The Ca^2+^ was calculated using the Maxchelator program from Stanford University. Images were acquired with a Leica (SP5) confocal microscope. Excitation was achieved by a white light laser fitted at 552 nm and emission collected at 575 nm. Analyses were made with Image J program.

### FRET measurements of cAMP levels

FRET imaging experiments were performed 48 h after infection of neonatal cardiomyocytes. Cells were bathed in Hepes-buffered Ringer's solution containing: 125 mM NaCl, 25 mM HEPES, 10 mM glucose, 5 mM K_2_HPO_4_, 1 mM MgSO_4_ and 1 mM CaCl_2_, pH 7.4. For sAC activation by HCO_3_^−^, the medium was the Krebs–Henseleit solution containing: 120 mM NaCl, 2.09 mM K_2_HPO_4_, 0.34 mM KH_2_PO_4_, 24 mM NaHCO_3_, 1 mM MgSO_4_, 1 mM CaCl_2_ and 10 mM d-glucose. Krebs–Henseleit solution was gassed continuously with 95% O_2_/5% CO_2_ to maintain a pH of 7.4.^35^ Real-time FRET experiments were performed at room temperature. Images were captured every 5 s using the × 40 oil-immersion objective of an inverted microscope (Nikon, Champigny sur Marne, France) connected to a Cool SNAP HQ2 camera (Photometrics, Tucson, AZ, USA) controlled by the Metafluor software (Molecular Devices, Sunnyvale, CA, USA). The donor (mTurquoise2)^36^ was excited during 300 ms by a xenon lamp (Nikon) using a 440/20BP filter and a 455LP dichroic mirror. Dual-emission imaging of donor and acceptor was performed using a dual-view emission splitter equipped with a 510 LP dichroic mirror and BP filters 480/30 and 535/25 nm, respectively.

### Data analysis

Results are expressed as mean±S.E.M. The Origin software (Northampton, MA, USA) was used for statistical analysis. Differences between groups have been analyzed by one-way ANOVA and Student's *t*-test. A value of *P*<0.05 were considered as statistically significant. The number of animals, cells and independent experiments performed is indicated in the figure legends.

## Abbreviations

2HE: 2-hydroxyestradiol
AC: adenylyl cyclase
ANT: adenine nucleotide translocase
CCCP: carbonyl cyanide m-chlorophenyl hydrazone
CsA: cyclosporine A
ΔΨm: mitochondrial membrane potential
Epac: exchange protein directly activated by cAMP
HCO_3_^−^: bicarbonate
HF: heart failure
IM: inner membrane
MCU: mitochondrial calcium uniporter
mNCX: mitochondrial Na^+^/Ca^2+^ exchanger
MPT: mitochondrial permeability transition
OM: outer membrane
PKA: protein kinase A
ROS: reactive oxygen species
sAC: soluble adenylyl cyclase
sAC_fl_: full-length soluble adenylyl cyclase
sAC_t_: truncated soluble adenylyl cyclase
