# Supplementary material for: A cardiac mitochondrial cAMP signaling pathway regulates calcium accumulation, permeability transition and cell death
Source: Cell Death Dis. 2016 Apr 21;7(4):e2198–. doi: 10.1038/cddis.2016.106 (PMC4855650; doi:10.1038/cddis.2016.106)
Supplement: Supplementary Information [file cddis2016106x1.pdf]

A cardiac mitochondrial cAMP signaling pathway regulates calcium accumulation, permeability transition and cell death

Wang et al.

Supplemental methods

Mitochondrial transmembrane potential measurement in adult cardiomyocytes

Mitochondrial transmembrane potential ($\Delta\Psi m$) was measured according to the manufacturer's instructions using Mito-ID® (Enzo Life Sciences, Villeurbanne, France). The orange fluorescence emission (Excitation = 540 nm, Emission = 570 nm) associated with energized mitochondria with high transmembrane potential and the green fluorescence emission (Excitation = 485 nm, Emission = 530 nm) associated with depolarized mitochondria with low transmembrane potential were recorded. The images were acquired under a 63×Plan-Apochromat (NA 1.40) oil immersion objective lens using a confocal microscope (Leica SP8).

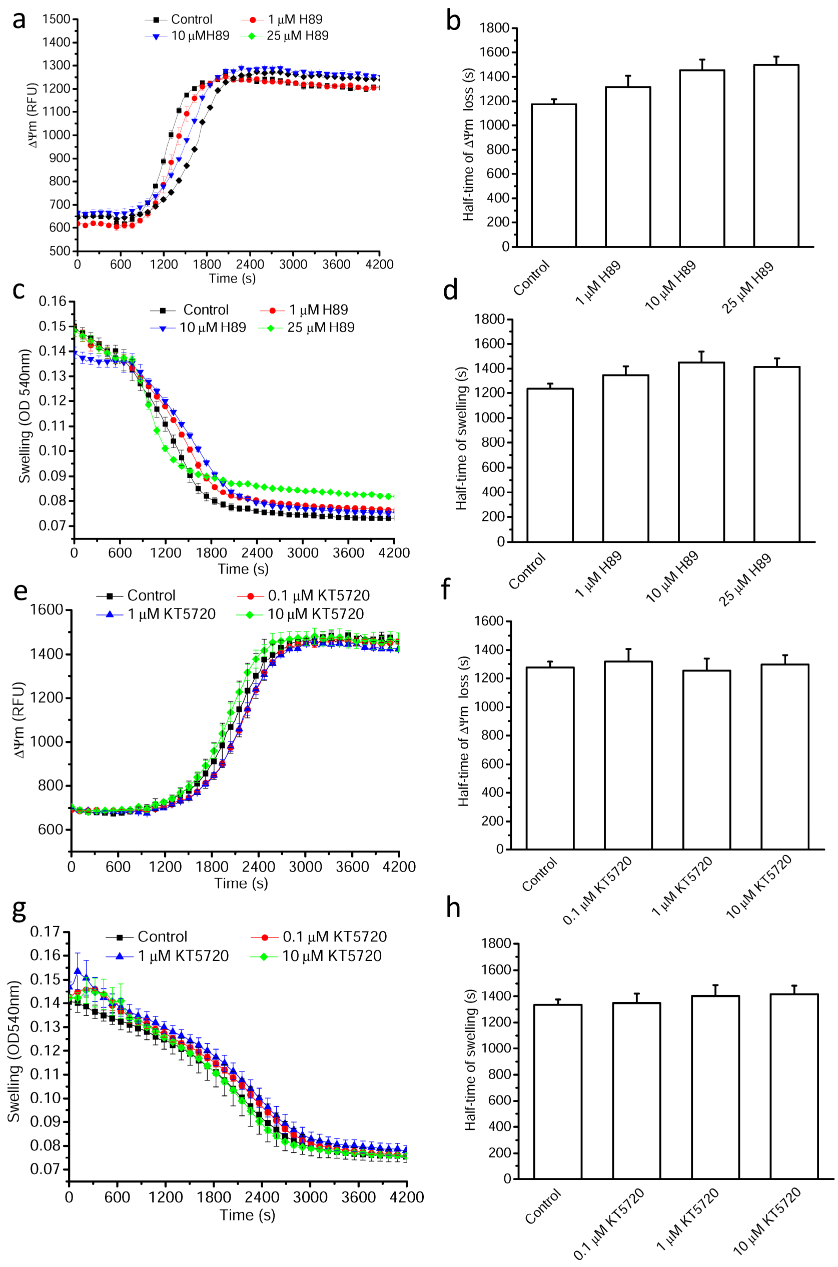

Supplemental Figure1. PKA is not involved in the Ca^{2+} **effects on mitochondrial membrane potential and swelling. (a)** Effect of H89 (1 μM, 10 μM and 25 μM) on ΔΨm loss induced by 10 μM Ca^{2+}. **(b)** Half-time of ΔΨm loss induced by 10 μM Ca^{2+} calculated from experiments such as shown in (**a**). **(c)** Effects of H89 (1 μM, 10 μM and 25 μM) on mitochondrial matrix swelling induced by 10 μM Ca^{2+}. **(d)** Half-time of mitochondrial swelling induced by 10 μM Ca^{2+} calculated from experiments such as shown in (**c**). **(e)** Effect of KT5720 (0.1 μM, 1 μM and 10 μM) on ΔΨm loss induced by 10 μM Ca^{2+}. **(f)** Half-time of ΔΨm loss induced by 10 μM Ca^{2+} calculated from experiments such as shown in (**e**). **(g)** Effect of KT5720 (0.1 μM, 1 μM and 10 μM) on mitochondrial matrix swelling induced by 10 μM Ca^{2+}. **(h)** Half-time of mitochondrial swelling induced by 10 μM Ca^{2+} calculated from experiments such as shown in (**g**) (n=3-4).

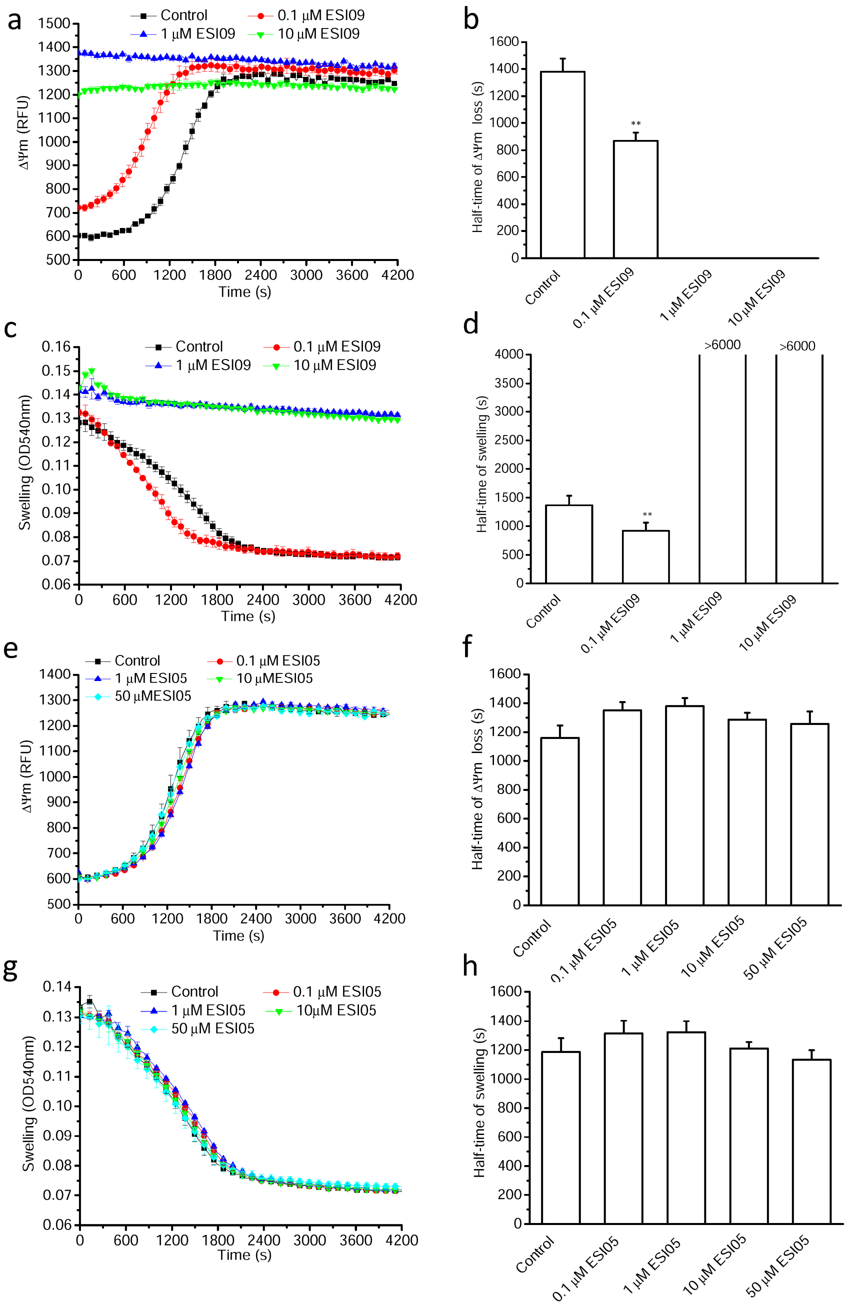

Supplemental Figure 2. Epac2 is not involved in the Ca^{2+} **effects on mitochondrial function. (a)** Effect of ESI09 (0.1 μM, 1 μM and 10 μM) on ΔΨm loss induced by 10 μM Ca^{2+}. **(b)** Half-time of ΔΨm loss induced by 10μM Ca^{2+} calculated from experiments such as shown in (**a**). **(c)** Effects o of ESI09 (0.1 μM, 1 μM and 10 μM) on mitochondrial matrix swelling induced by 10 μM Ca^{2+}. **(d)** Half-time of mitochondrial swelling induced by 10 μM Ca^{2+} calculated from experiments such as shown in (**c**). **(e)** Effect of ESI05 (0.1 μM, 1 μM, 10 μM and 25 μM) on ΔΨm loss induced by 10 μM Ca^{2+}. **(f)** Half-time of mitochondrial swelling induced by 10 μM Ca^{2+} calculated from experiments such as shown in (**e**). **(g)** Effect of ESI05 (0.1 μM, 1 μM, 10 μM and 25 μM) on mitochondrial matrix swelling induced by 10 μM Ca^{2+}. **(h)** Half-time of mitochondrial swelling induced by 10 μM Ca^{2+} calculated from experiments such as shown in (**g**). ***P*<0.01(n=3-9).

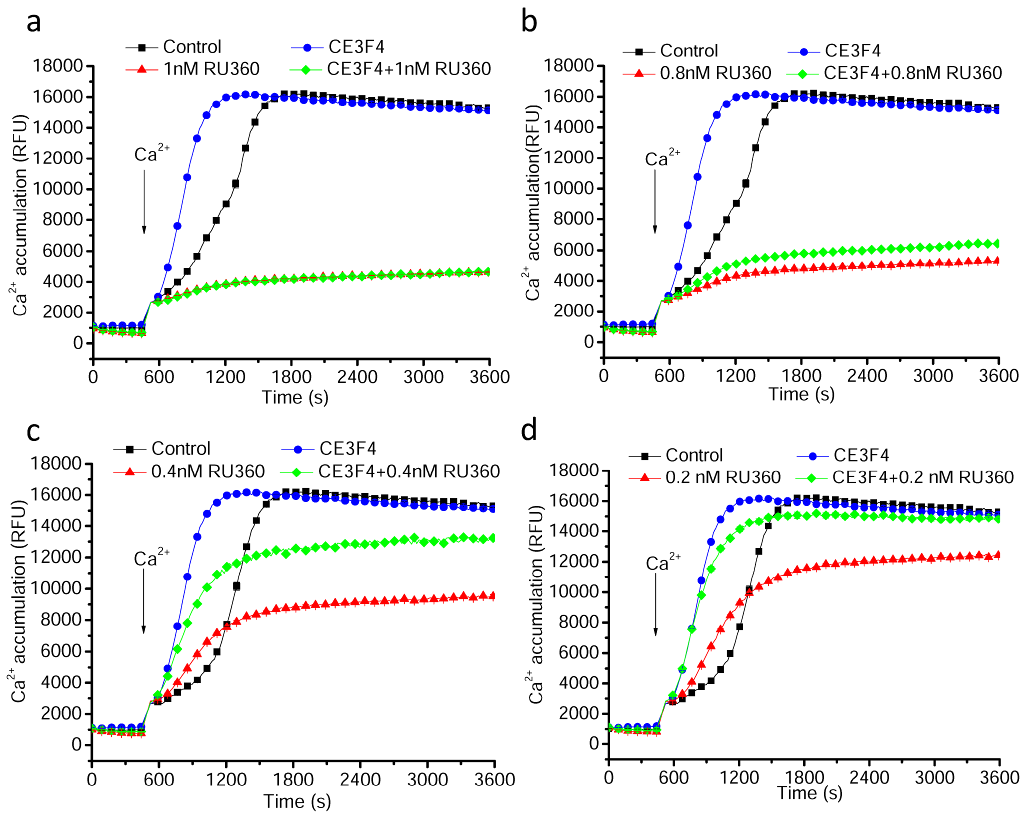

Supplemental Figure 3. Effect of RU360 and CE3F4 on mitochondrial Ca^{2+} levels measured with Rhod-2 in isolated mitochondria. (a) Measurement of Ca^{2+} accumulation in isolated mitochondria using 2 µM Rhod-2 in the absence or presence of 50 µM CE3F4, 1 nM RU360 or both. **(b)** Measurement of Ca^{2+} accumulation in isolated mitochondria using 2 µM Rhod-2 in the absence or presence of CE3F4, 0.8 nM RU360 or both. **(c)** Measurement of Ca^{2+} accumulation in isolated mitochondria using 2 µM Rhod-2 in the absence or presence of CE3F4, 0.4 nM RU360 or both. **(d)** Measurement of Ca^{2+} accumulation in isolated mitochondria using 2 µM Rhod-2 in the absence or presence of CE3F4, 0.2 nM RU360 or both (n=3).

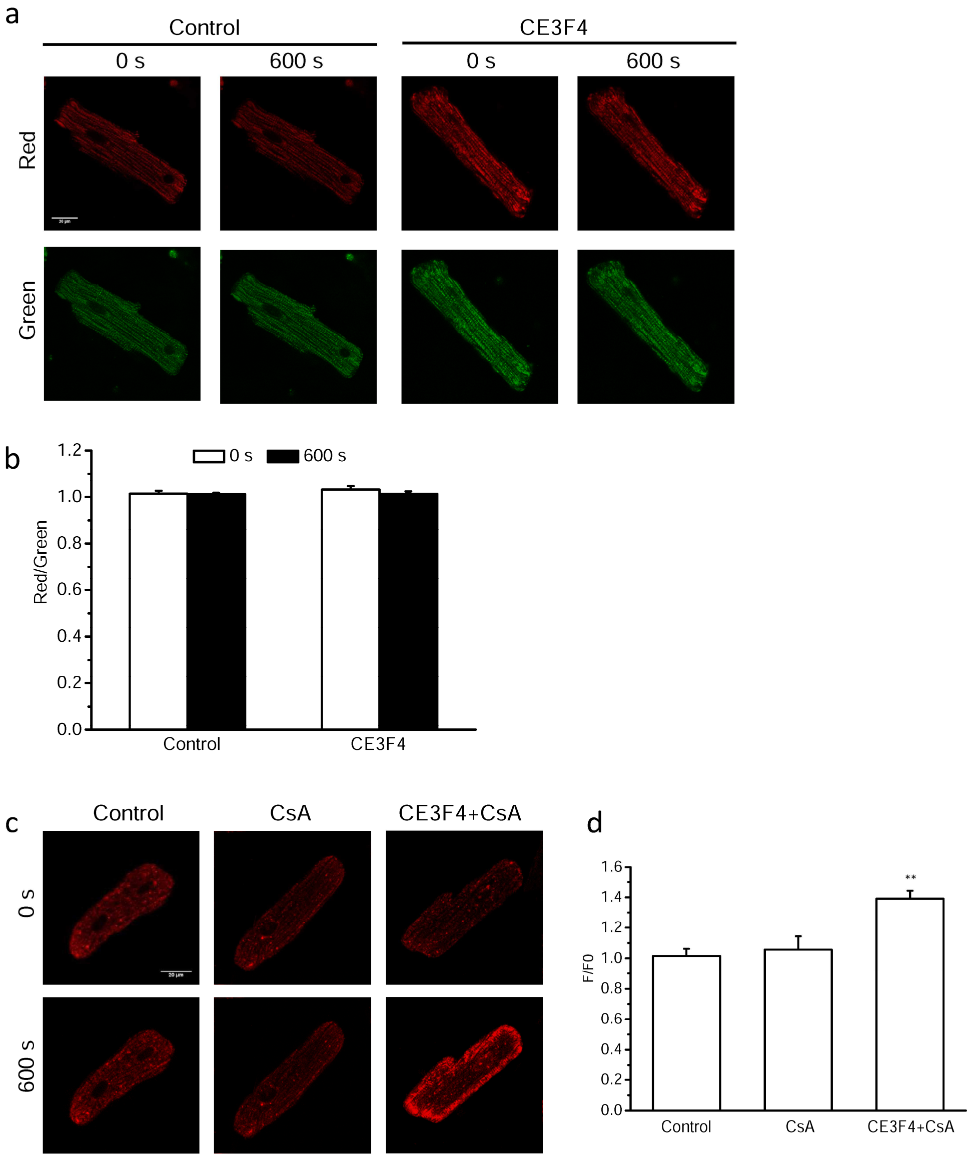

Supplemental Figure 4. Ca^{2+} concentrations did not affect ΔΨm and did not involve the PTPC. (a) Confocal images of adult rat cardiomyocytes showing JC10 fluorescence in two emissions channels 525 nm (green images) and 590 nm (red images). The green fluorescence shows depolarized mitochondria (monomeric probe), whereas red fluorescence shows energized mitochondria (aggregated probe). Cardiomyocytes were labeled with JC10 for 15 min then permeabilized with 5 µg/mL digitonin. Then the cells were exposed to 50 µM CE3F4 or vehicle for 10 min and 200 nM free Ca^{2+} was applied at time 0 s. Bar scale, 20 µm. **(b)** Red to green ratio of JC10 measured from confocal images such as shown in (**a**)(n=6). **(c)** Representative recording of confocal images of Rhod-2-labeled cardiomyocytes at time 0 s and 600 s. Cells were sequentially permeabilized with digitonin in Ca^{2+} free internal solution, treated with 50 µM CE3F4, CE3F4 + 1 µM CsA or vehicle for 10 min, then 200 nM Ca^{2+} was applied. Bar scale, 20 µm. **(d)** Mean changes in Rhod-2 signal (n=9). ***P*<0.01.

a

Anatomical parameters measured in sham and HF rats.

	Sham (n=4)	HF (n=4)	*P**
Body weight (g)	594±7	559±26	NS
Heart weight (g)	1.66±0.04	3.6±0.23	<0.001
Lung weight (g)	1.67±0.04	4.29±0.59	<0.01
Tibia length (cm)	4.39±0.05	4.35±0.08	NS
Heart weight/body weight (mg/g)	2.79±0.05	6.45±0.31	<0.001
Lung weight/body weight (mg/g)	2.82±0.09	7.56±0.80	<0.01
Heart weight/tibia length (g/cm)	0.37±0.01	0.82±0.04	<0.001
Lung weight/tibia length (g/cm)	0.38±0.01	0.98±0.12	<0.01

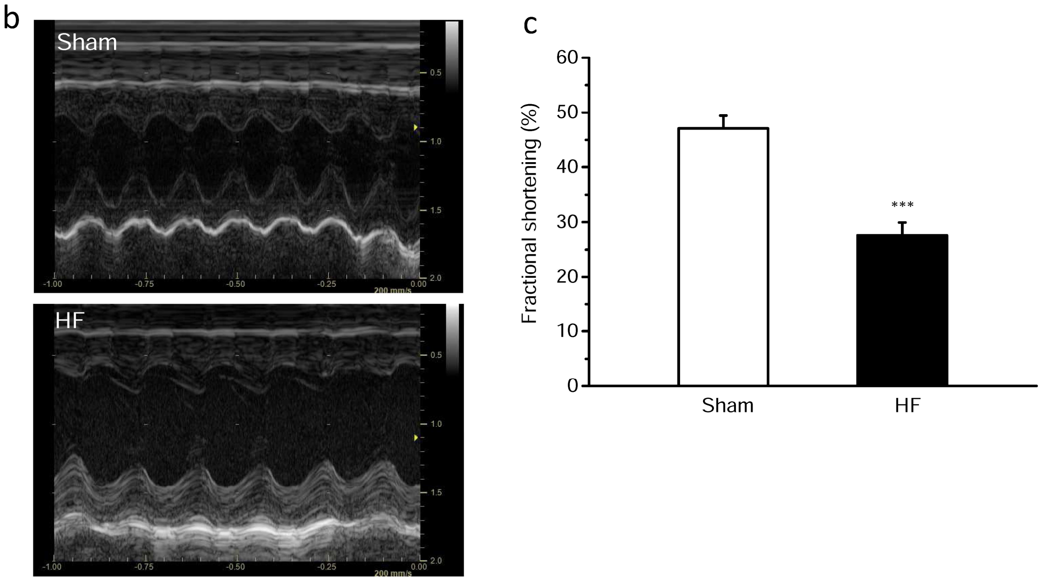

Supplemental Figure 5. Anatomical parameters and echocardiographic analysis of Sham and HF rat. (a) Anatomical parameters measured in sham and HF rats.Values are mean ± SEM; n= number of animals. * Statistical differences between heart failure (HF) rats and sham values; NS: not significant. HF was induced by transverse aortic constriction (TAC) as described in material and methods. **(b)** Representative echocardiographic images from Sham and HF rat hearts. **(c)** Fractional shortening in Sham and HF rat hearts. *** *P*<0.001 (n=4).

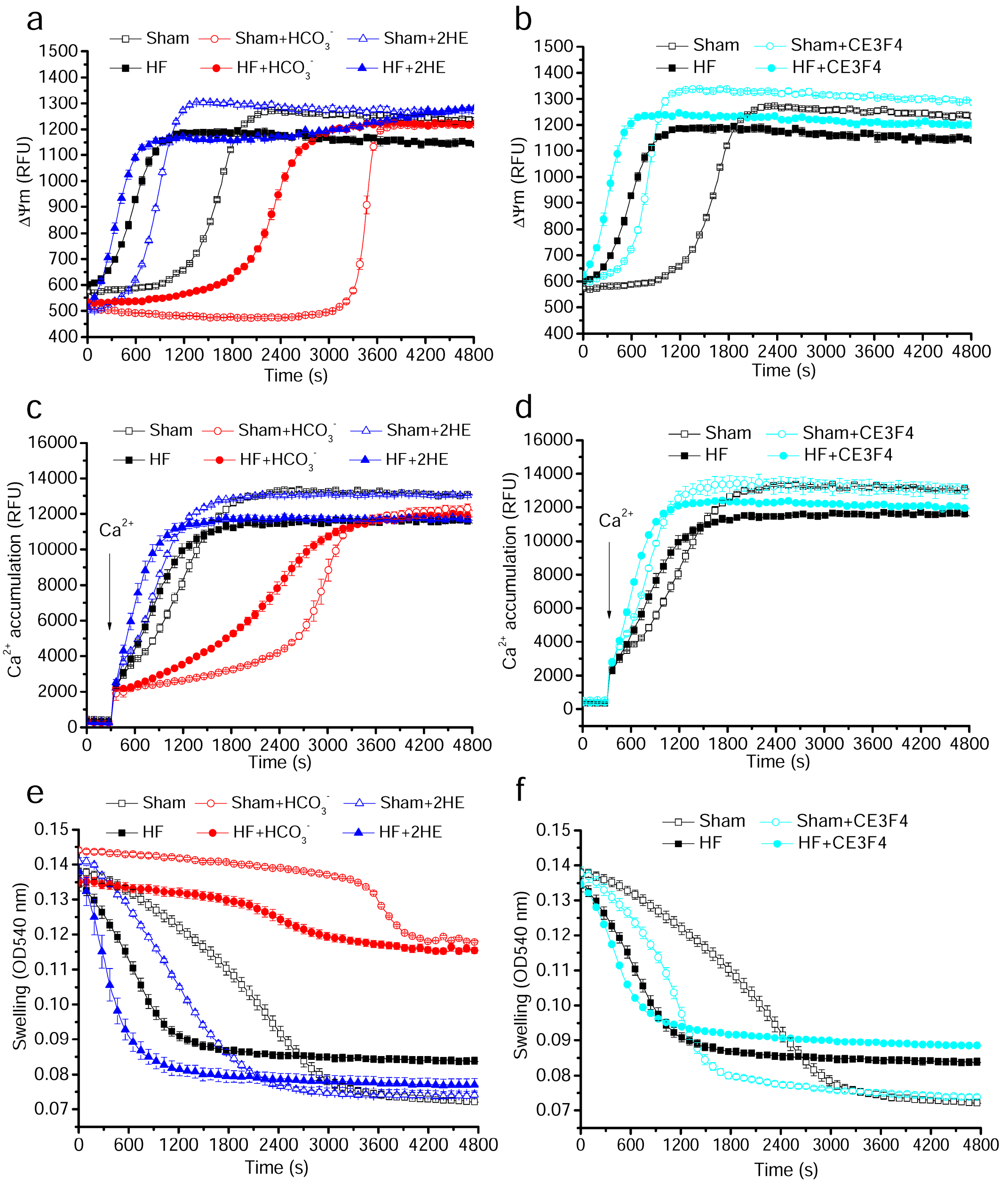

Supplemental Figure 6. cAMP regulation of mitochondrial membrane potential and Ca^{2+} uptake are preserved in HF rats. Evaluation of ΔΨm in Sham and HF cardiac isolated mitochondria induced by 10 µM Ca^{2+} in response to sAC stimulation by 15 mM HCO_3^- and sAC inhibition by 25 µM 2HE **(a)** and Epac1 inhibition by 50 µM CE3F4 **(b)**. Mitochondrial Ca^{2+} accumulation following addition of 10 µM Ca^{2+} in response to sAC stimulation by 15 mM HCO_3^- and sAC inhibition by 25 µM 2HE **(c)** and Epac1 inhibition by 50 µM CE3F4 **(d)**. Evaluation of mitochondrial swelling in Sham and HF cardiac isolated mitochondria induced by 10 µM Ca^{2+} in response to sAC stimulation by 15 mM HCO_3^- and sAC inhibition by 25 µM 2HE **(e)** and Epac1 inhibition by 50 µM CE3F4 **(f)**. Panels (a-f) display one experiment representative of 4 experiments done in triplicate.
